# Gene SH3BGRL3 regulates acute myeloid leukemia progression through circRNA_0010984 based on competitive endogenous RNA mechanism

**DOI:** 10.3389/fcell.2023.1173491

**Published:** 2023-06-12

**Authors:** Xiancong Yang, Yaoyao Wang, Simin Rong, Jiayue An, Xiaoxu Lan, Baohui Yin, Yunxiao Sun, Pingyu Wang, Boyu Tan, Ye Xuan, Shuyang Xie, Zhenguo Su, Youjie Li

**Affiliations:** ^1^ Department of Clinical Laboratory, The Second Medical College of Binzhou Medical University, Yantai, China; ^2^ Department of Biochemistry and Molecular Biology, Binzhou Medical University, Yantai, China; ^3^ Department of Pediatrics, Yantai Affiliated Hospital of Binzhou Medical University, Yantai, China

**Keywords:** Acute myeloid leukemia, TCGA, SH3BGRL3, Hsa_circ_0010984, qRT-PCR

## Abstract

**Introduction:** Acute myeloid leukemia (AML) is a malignant proliferative disease affecting the bone marrow hematopoietic system and has a poor long-term outcome. Exploring genes that affect the malignant proliferation of AML cells can facilitate the accurate diagnosis and treatment of AML. Studies have confirmed that circular RNA (circRNA) is positively correlated with its linear gene expression. Therefore, by exploring the effect of SH3BGRL3 on the malignant proliferation of leukemia, we further studied the role of circRNA produced by its exon cyclization in the occurrence and development of tumors.

**Methods:** Genes with protein-coding function obtained from the TCGA database. we detected the expression of SH3BGRL3 and circRNA_0010984 by real-time quantitative polymerase chain reaction (qRT-PCR). We synthesized plasmid vectors and carried out cell experiments, including cell proliferation, cell cycle and cell differentiation by cell transfection. We also studied the transfection plasmid vector (PLVX-SHRNA2-PURO) combined with a drug (daunorubicin) to observe the therapeutic effect. The miR-375 binding site of circRNA_0010984 was queried using the circinteractome databases, and the relationship was validated by RNA immunoprecipitation and Dual-luciferase reporter assay. Finally, a protein‐protein interaction network was constructed with a STRING database. GO and KEGG functional enrichment identified mRNA-related functions and signaling pathways regulated by miR-375.

**Results:** We identified the related gene SH3BGRL3 in AML and explored the circRNA_0010984 produced by its cyclization. It has a certain effect on the disease progression. In addition, we verified the function of circRNA_0010984. We found that circSH3BGRL3 knockdown specifically inhibited the proliferation of AML cell lines and blocked the cell cycle. We then discussed the related molecular biological mechanisms. CircSH3BGRL3 acts as an endogenous sponge for miR-375 to isolate miR-375 and inhibits its activity, increases the expression of its target YAP1, and ultimately activates the Hippo signaling pathway involved in malignant tumor proliferation.

**Discussion:** We found that SH3BGRL3 and circRNA_0010984 are important to AML. circRNA_0010984 was significantly up-regulated in AML and promoted cell proliferation by regulating miR-375 through molecular sponge action.

## Introduction

With high heterogeneity and invasive ability, acute myeloid leukemia (AML) is a malignant proliferative disease affecting the hematopoietic system (Jie and Xiaochun, 2021). Many studies have claimed that circular RNA (circRNA) is an important regulator involved in AML progression. Developments in molecular targeted therapy techniques and cell transplantation have significantly improved the conditions of most patients with AML. However, many patients still relapse and have poor long-term prognosis (Azmi and Balasubramanian, 2021; Xiaodan et al., 2021). Therefore, exploring genes that affect and improve the prognosis of AML is necessary to developing effective monitoring and treatment options.

The post-translational modification of SH3 domain-binding glutamate-rich protein 3 (SH3BGRL3) was identified as tumor necrosis factor-α (TNF-α) inhibitory protein TIP-B1. SH3BGRL3 encodes a highly conserved small protein composed of 93 amino acids. A study showed that the SH3BGRL3 gene expression was upregulated in HL60 cells, which was related to the differentiation induced by phorbol myristate acetate. These results indicated that SH3GRL3 plays a role in the differentiation-related signal transduction pathway network (Shuhong et al., 2005). The expression of some circRNA molecules has a certain degree of coexpression with host linear genes, such as the positive correlation between gene PDK1 and circPDK1 expression levels (Jiewei et al., 2022). Therefore, we decided to study the circRNA produced by the cyclization of SH3BGRL3 exons.

CircRNA is an emerging reference tool for the diagnosis and treatment of various hematological malignancies. It is a product of closed reverse splicing of precursor mRNA. The disorder of a circRNA is significantly correlated with the malignancy and drug resistance of cancer and has reliable detection potential in plasma, saliva, and other samples. In addition, circRNA and host genes have a certain degree of correlation. For example, the interactions of circRNA molecules with sites in host genes can repair DNA damage and increase cell sensitivity to drug toxicity (Gao et al., 2020; Junhong et al., 2022; Kjems, 2022; Zhang, 2022). Most circRNA molecules consist of different numbers of exons, are covalently closed RNA molecules produced by the reverse splicing of pre-mRNA molecules, have no 5′ caps and 3′ tails, are extremely stable, and have cell- or tissue-specific expression patterns (Ming et al., 2022). circRNA is involved in tumorigenesis due to its biological characteristics and through a variety of mechanisms, such as microRNA (miRNA) sponge, RNP binding, and translation template, and plays a potential regulatory role in a variety of cancer diseases (cardiovascular diseases, lung cancer, and leukemia) (Jie et al., 2022; Qidong et al., 2022; Zhang et al., 2022). In addition, circRNA affects tumor progression by regulating autophagy (Miao et al., 2022). circRNA has been reported in many human malignancies, including liver, lung, and bladder cancer. circRNA_0079593 can promote the proliferation, metastasis, and glucose metabolism of melanoma by regulating the miR-516b/GRM3 axis (Jiajing and Lu, 2020). circ_POLA2 can promote the proliferation of AML cells by regulating miR-34a (Wang and Zhu, 2021). circRNA_0010984 has not been reported in the literature.

## Materials and methods

### Patients and samples

Diagnostic peripheral blood samples were collected from 13 patients with AML (16–63 years) and 13 healthy controls at Binzhou Medical University Hospital between October 2021 and November 2022. Normal and tumor blood samples were stored at −80°C until RNA extraction. Detailed information about these patients has been added to the Notes form. The study was approved by the ethics committee of Binzhou Medical University according to the principles of the Helsinki Declaration.

### Data download and identification

Sample information of the TCGA database included 136 patients with leukemia and 15 sample controls. The DESeq2 package of R software was used in screening differentially expressed mRNA (DEmRNA) in AML and selecting DEmRNA (|log2FC| > 1, padj <0.05). The GEOquery package of R software was used in obtaining the GSE193094 microarray data set of the GEO database, and the grouping was designated as parental HL-60, Ara-C resistant (HRA) and DNR-resistant (HRD). The limma package of R software was used for data correction and gene expression analysis. Construction and Functional Enrichment of Competitive Endogenous Regulatory RNA Network CircRNA–miRNA regulatory sites were queried using circinteractome (http://circinteractome.nia.nih.gov), and miRNA–mRNA regulatory sites were queried using Targetscan (www.targetscan.org), microT (www.biostars.org). Commonly recognized mRNA molecules in these miRNA-related databases were obtained. A visual regulatory network was constructed using cytoscape (version v3.9.0). Commonly identified hub genes were used for GO and KEGG functional enrichment analyses, and the clusterprofiler plug-in of R software was used in obtaining biological pathways.

### Weighted gene coexpression network analysis

Gene modules can be detected, and the correlation between each module and patient survival characteristics can be evaluated using a constructed a weighted gene coexpression network (Lin, 2022). We performed data processing on the information of 136 patients leukemia from the TCGA database for WGCNA analysis. The soft threshold (β value) was set at 9, and then the adjacency matrix of the sample was transformed into a topological overlap matrix. After merging and clustering similar modules, we sorted the module feature matrix, and the correlation between the model feature and sample information matrices was calculated. The above correlation matrix and *p* values were visualized using a labeled heatmap plug-in.

### Cell culture and processing

AML cell lines THP-1, HL-60, and U937 and human embryonic kidney cell line HEK-293T were provided by Cell Bank of Chinese Academy of Sciences (Shanghai, China). Daunorubicin (DAU; Aladdin Industries) was added to HL-60 cells at the logarithmic growth phase and U937 cell suspension at different final concentrations. Drug dose was gradually increased during the drug action period, and the optimal drug concentration (IC50) that affected the inhibition of cells at 50% survival in the drug culture system after 24 h was finally screened. The cells were cultured in 10% fetal bovine serum (all from Gibco; Thermo Fisher Scientific) and 1% penicillin–streptomycin (Beyotime Institute of Biotechnology) in RPMI 1640 medium or DMEM in a humidified incubator (37°C, 5% CO2).

### Real-time polymerase chain reaction

Blood cells and serum were separated from peripheral blood samples, and a threefold volume of red blood cell lysate (Biosharp Biotechnology) was added to the extracted blood cells. The mixture was gently mixed, allowed to stand for 15 min, and centrifuged at 2,000 rpm for 5 min. Total RNA was extracted from leukocyte precipitation with TRIzol reagent (Thermo Fisher Scientific). We first used a PrimeScript RT kit (Takara Bio) to reverse-transcribed RNA to produce cDNA, which was then determined by real-time quantitative polymerase chain reaction (qRT-PCR) with a real-time fluorescent quantitative PCR system. The qPCR conditions were as follows: initial denaturation at 95°C for 3 min; 40 cycles, 95°C for 20 s, annealing at 60°C for 20 s, extension at 72°C for 20 s. Then, 2−ΔΔ Cq was calculated using relative expression and fold change. Primers were purchased from Guangzhou Ruibo Biological Co., Ltd. The sequences of the primers were as follows: SH3BGRL3, forward 5′-ACA​TCT​CCC​AGG​ACA​ACG-3′ and reverse 5′-TCC​ACA​GCC​TCC​ACG​AA-3′; circSH3BGRL3, forward 5′-TCC​ATT​GGC​AAT​CAA​GTC​C-3′ and reverse 5′-AAG​GCT​CGC​ATC​TCA​TCC-3′; GAPDH forward 5′-GCT​GAA​CGG​GAA​GCT​CAC​TG-3′ and reverse 5′-GTG​CTC​AGT​GTA​GCC​CAG​GA-3′; miR-375-3p, forward 5′-TTT​GTT​CGT​TCG​GCT​CGC​GTG​A-3′ and reverse 5′-CGA​ACA​TGT​ACA​GTC​CAT​GGA​TAG-3′; U6, forward 5′-CTC​GCT​TCG​GCA​GCA​CA-3′ and reverse 5′-AAC​GCT​TCA​CGA​ATT​TGC​GT-3′. GAPDH and U6 were used as reference genes. Sanger sequencing was performed to validate the sequence of hsa_circ_0010984.

### RNA R assay and actinomycin D

Total RNA (5 µg) was exposed to 1–3 U/µg RNA RNase R (20 U/µL) at 37°C for 30 min, and water without RNase was used as the control (Mock). The total volume of the reaction system was 20 μL, and the internal reference in the RNase R-group was used as the calculation standard. After RNase R digestion, RT-PCR was performed directly, and the enzyme was inactivated at 70°C for 10 min. For actinomycin D experiment, AML cells were exposed to 2 μg/mL actinomycin D for 4, 8, 12, and 24 h. The cells were then harvested, and the stability of circSH3BGRL3 and SH3BGRL3 was analyzed using qRT-PCR.

### Small interfering RNAs (siRNAs), vector construction

Small interfering RNAs (siRNAs) against circ_0010984 and the negative control RNA duplex (siRNA-NC) were purchased from RiboBio, Guangzhou, China. For the overexpression vector, Front circular Frame (2,278–2,510 bp) and Back circular Frame (3,149–3,400 bp) were added to the circRNA fragment sequence. We used PCR amplification, cloned into plasmid vector (plv-circRNA). The PCR conditions were as follows: initial denaturation at 95°C for 1 min; 30 cycles of 95°C for 20 s, 60°C annealing for 20 s and extension at 72°C for 20 s. The amplification was completed with a final step of 72°C for 5 min. PCR products were separated by electrophoresis on a 1% agarose gel followed by visualization under the Tanon 2,500 gel imaging system (Tanon Science and Technology Co., Ltd.). Circbase ()was” title = "http://www.circbase.org/)was">http://www.circbase.org/)was used to analyze the sequence of circular RNA.

### Lentivirus packaging and cell infection

The 293T cells were prepared, and the cell density reached 80%–90% 24 h before transfection, and the cells were starved for 1 h. The objective plasmid:PSPAX2 plasmid:MD2G plasmid (7.5:5.5:2 μg; v = m/c) was diluted with normal saline to a volume of 500 µL and placed at room temperature for 5 min. PEI (45 µL) was prepared, diluted with normal saline to a volume of 500 μL, mixed, and transfected into a 293T cell culture. The virus supernatant was collected and filtered with a 0.45 µm filter. The supernatant was centrifuged at 8,000 g for 30 min at 4 °C for the removal of cell debris. HL-60 and U937 cells were seeded in six-well plates and cultured in a 5% CO2 incubator at 37 °C until the cell density was 50%–60%. Lentivirus infection was performed, and the liquid was replaced after 6–8 h for subsequent experiments. After 24 h, changes in GFP expression were detected by fluorescence microscopy and flow cytometry, and the images were captured. For conventional cell transfection, we used a Lipofectamine 2000 transfection reagent (liposome 2000: siRNA = 1:1, liposome 2000: plasmid = 1:1, mimic final concentration = 20 nM).

### Cell viability assay

Cell viability was measured by a cell counting kit-8 (CCK-8). HL-60 and U937 cells were seeded in 96-well plates (4 × 103 cells/well) and cultured at 37°C in an incubator. CCK-8 (10 μL; Beyotime Institute of Biotechnology) was added to the well at 0, 24, 48, and 72 h for 2 h. Absorbance at 450 nm was measured by a microplate reader.

### Cell cycle analysis

The cells were washed with PBS, centrifuged at 1,500 rpm for 3 min, collected, and adjusted to a cell concentration of 1 × 106/mL. Then, 1 mL of single cell suspension was collected. After centrifugation, the supernatant was removed, and 1 mL of 70% precooled ethanol was added to the cells for 2 h of fixation overnight. The samples were stored in a refrigerator at 4°C. The fixative was washed before staining, and 100 µL of LRNaseA solution was added to the cell precipitate and placed in a 37°C water bath for 1 h. Approximately 400 µL of PI staining solution was mixed, subjected to ta temperature of 4°C away from light incubation for 1 h. Flow cytometry detection was performed at a wavelength of 488 nm (red fluorescence).

### Cell differentiation

THP-1 cells were treated with 50 ng/mL phorbol myristate 13-acetate for 24 h, and the morphology of cells was observed. LPS (100 ng/mL) was added and incubated for 48 h. Wright–Giemsa staining was performed after 48 h. Finally, monocyte differentiation was assessed by observing cell morphology on the slides and detecting macrophage surface molecular marker CD86(FACS analysis). Cells were stained with anti-human CD86-PE (eBioscience, Carlsbad, CA, United States) monoclonal antibodies. The data were analyzed using FlowJo software.

### RNA-binding protein immunoprecipitation

The interaction between circRNA_0010984 and miR-375 was determined with an RIP assay kit (Guangzhou Geneseed Biotech Co., Ltd.). After sample pretreatment according to this protocol, leukemia cells THP-1, HL-60, and U937 were lysed. After pretreatment with magnetic beads, they were ligated with antibodies for 2 h, and IP (capture antigen) was reacted at 4°C for 2 h overnight. The experimental group used human Argonaute 2 (Ago2) and mouse immunoglobulin G (IgG) as negative controls. After the RNA-binding protein was eluted and purified, the coprecipitated RNA was extracted, and the expression was detected by qRT-PCR.

### Luciferase activity assay

Wild type (WT) and mutant (Mut) fragments of circ_0010984 containing miR-375 binding sites were amplified and inserted into pmiR-GLO vector to generate recombinant plasmids WT-pmiR-GLO-circ_0010984, Mut-pmiR-GLO-circ_0010984. For the dual-luciferase reporter assay, Cancer cells (1 × 105 cells/well) were plated into 6-well plates and were cotransfected with WT-circ_0010984, or Mut-circ_0010984 and miR-375 mimics or scramble control. The luciferase activity was detected according to the manufacturer’s instructions using a double luciferase detection kit (Vazyme).

### Protein imprinting analysis

The transfected cells were collected, washed twice with cold PBS, and lysed on ice with 150 µL RIPA lysis buffer (Beyotime Biotechnology Institute) for 30 min. After each lane sample protein, 10% SDS-PAGE was used in separating the protein and transferred to a PVDF membrane. The membrane was blocked in 5% milk powder at 37°C for 2 h, and the primary antibody was incubated at 4°C overnight. The next day, the membrane was washed three times with TBST and incubated with goat anti-rabbit IgG (H + L) HRP at 4°C for 2 h for the capturing of the bands. ImageJ software was used in analyzing the density of the strip and measuring the gray value.

### Statistical analysis

R software (version 4.1.3) was used for single factor cox regression and Lasso regression analyses, and GraphPad Prism (version 8.0) software was used for processing receiver operating characteristic curve (ROC) analysis (Francis, 2022). The Kaplan-Meier method was used in evaluating the correlation between survival status and gene expression, and the survival curve was drawn. Log-rank test was used in evaluating significance. The control and experimental groups were compared using *t*-test. For unpaired samples, unpaired *t*-test was used. For paired samples, paired *t*-test was used. All calculations were performed using GraphPad Prism 8.0 and R software. In addition, Fisher exact test was performed using SPSS software. *p* < 0.05 was considered statistically significant.

## Results

### Characteristics of patients

**FIGURE 1:**
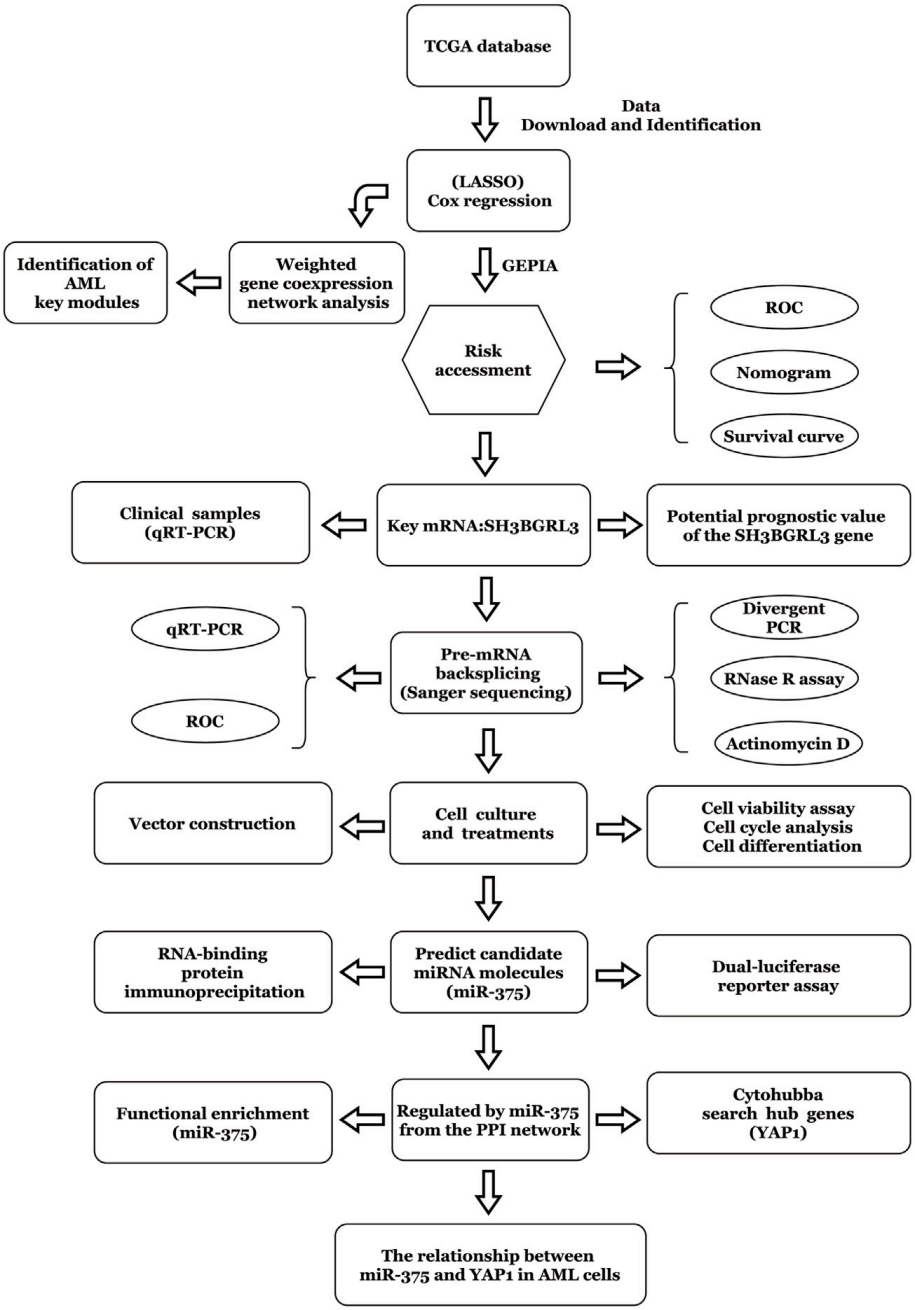
Method flow chart based on the construction of prognostic risk model.

The workflow chart of this study is shown in Figure 1. Patients without survival data or histopathological information were excluded.

### Screening of prognostic genes affecting acute myeloid leukemia

**FIGURE 2:**
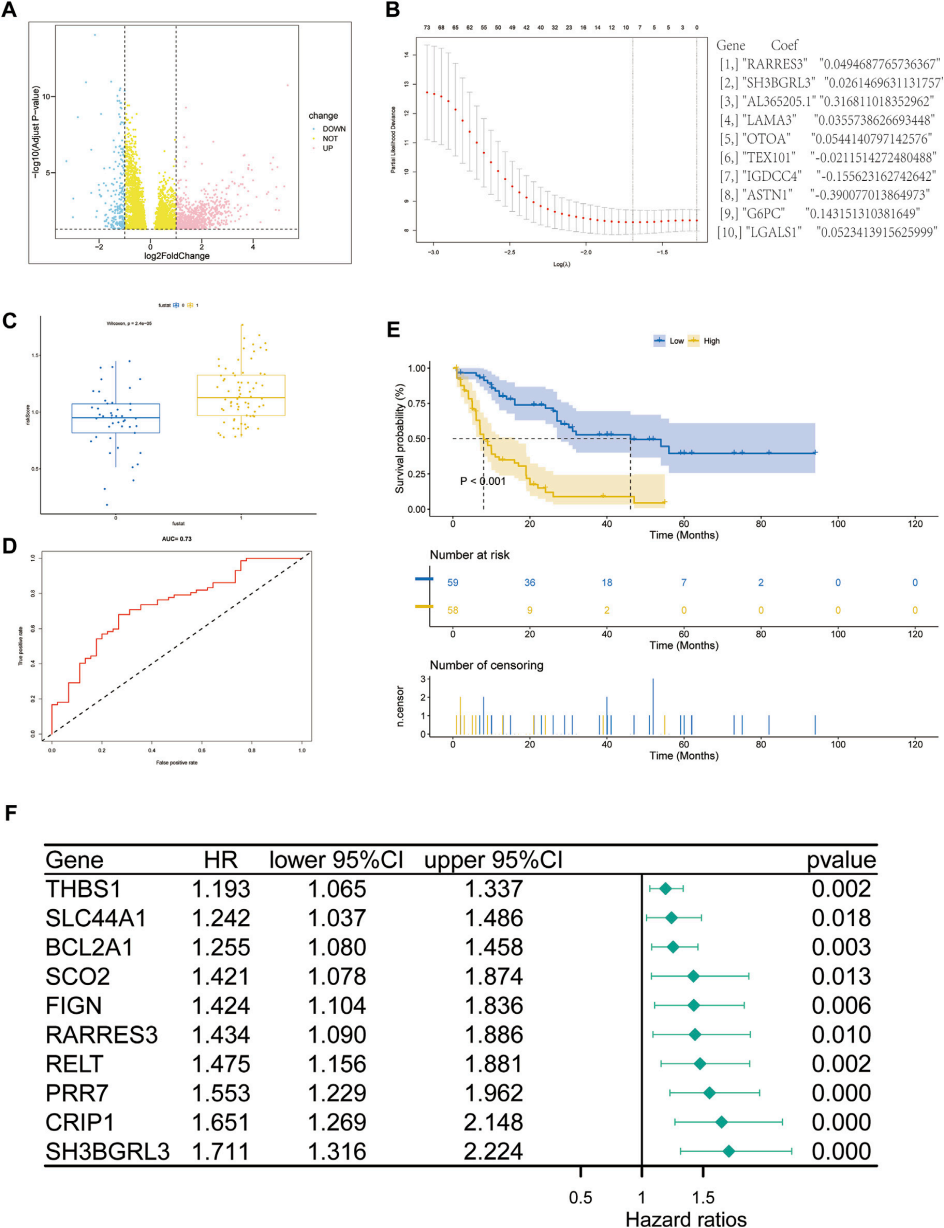
Screening of genes related to the prognosis of acute myeloid leukemia (AML): **(A)** Volcano map of differentially expressed gene mRNA (DEmRNA) from the TCGA database (*p*-value of differential genes is sorted from small to large). **(B)** Lasso coefficient spectrum of DEmRNA in AML prognosis. **(C,D)** ROC curve and scatter plot based on the risk model. The area under the curve (AUC) is 0.73. **(E)** The survival curve based on the risk model. **(F)** Forest plot to represent the COX regression model for evaluating AML prognosis-related genes.

A comprehensive analysis of RNA sequencing data of 151 TCGA gene-related expression from the UCSC Xena database, including 136 patients with leukemia and 15 related controls (Figure 2A). For the counts data, after log conversion (fpkm does not require log conversion), the R project DESeq2 package was used in analyzing the differential analysis of 19,620 protein-coding mRNA molecules (|log2FC| > 1, padj >1) DEGs. Subsequent Lasso regression model was constructed, the correlation coefficient of the prediction model was output, and the risk score was calculated. Ten genes related to prognostic risk (RARRES3, SH3BGRL3, AL365205.1, LAMA3, OTOA, TEX101, IGDCC4, ASTN1, G6PC, and LGALS1) were obtained (Figure 2B), and the risk score was calculated by multiplying the gene expression level by its corresponding regression coefficient (FanHongyi and Zhou, 2022). According to the median risk score, 117 AML samples containing prognostic information were divided into high-risk group (n = 58) and low-risk group (*n* = 59) (Figure 2C). A risk-related ROC curve was plotted using the ROCR package (Figure 2D). An area under the ROC curve (AUC) of 0.73 indicates that the risk score can be used in predicting a model (Zhiyuan et al., 2021). Survival analysis showed that the overall survival rate of patients in the high-risk group was significantly lower than that in the low-risk group (*P* 1, *p* < 0.05) (Figure 2E). The forest plot package was used in plotting the forest. Among the 10 genes shown in this model, SH3BGRL3 had a significant correlation with prognosis prediction (Figure 2F). Through multivariate regression analysis, we integrated multiple predictors. The risk assessment score was used in distinguishing high and low risk samples (Supplementary Figures S1A, B). The core gene SH3BGRL3 was selected for the establishment of a risk model nomogram. The total score of the nomogram showed that the gene SH3BGRL3 is useful in predicting the prognosis of AML. We used the GEO database to verify our conclusion (Supplementary Figure S1C).

### Construction of a weighted coexpression network and identification of disease key modules

**FIGURE 3:**
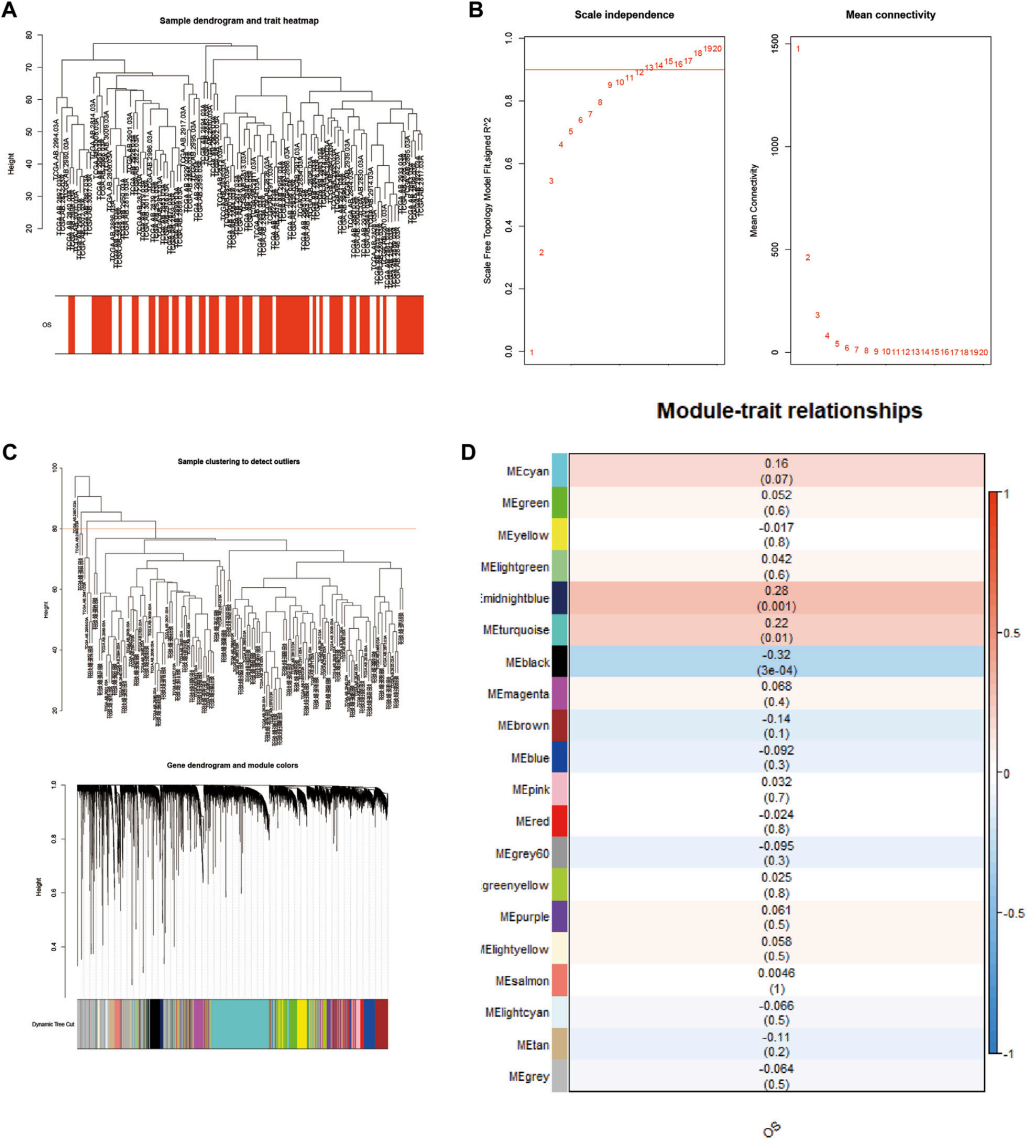
Construction of a weighted coexpression network, and identification of AML key modules: **(A)** Sample tree diagram and trait heat map based on TCGA database patient data. **(B)** Fitting index and power value scatter plot and average connectivity and power value scatter plot, view the best power value. **(C)** Based on the mRNA sample clustering tree from the TCGA database (clinical information is converted to color, white indicates low, red indicates high, and gray indicates missing). **(D)** Correlation between different modules and AML (correlation coefficients and *p* values are shown in the heat map).

To study the relationship between genes and survival characteristics, WGCNA analysis was performed to construct a weighted gene coexpression network, detect gene modules, and correlate gene modules with the survival status of AML patients (Yang, 2020). After clustering 136 patient samples from the TCGA database, the samples below the shear line were deleted and re-clustered, and a dendritic map was produced (Figure 3A). The function “sft $ powerEstimate” was used in estimating the best power value, and soft power of 9 was selected (Figure 3B). Then, using the TOM matrix gene clustering recognition dynamic detection module, we detected 20 modules (Figure 3C). After clustering and merging similar modules, the correlation between the model feature matrix and the sample information matrix is calculated, and the correlation map between the gene module and the clinical information is generated (Figure 3D). The correlation between gene module and clinical information showed that 11 modules, such as midnightblue module (*p* = 0.001, correlation coefficient = 0.28) and turquoise module (*p* = 0.01, correlation coefficient = 0.22), were positively correlated with patient death, and nine modules, such as MEblack module (*P* = 3e−04, correlation coefficient = −0.32), were negatively correlated with patient death. Notably, the gene SH3BGRL3 belongs to the turquoise module, suggesting that the SH3BGRL3 gene plays a key role in the prognosis of patients with AML.

### Potential prognostic value and related biological functions of the SH3BGRL3 gene

**FIGURE 4:**
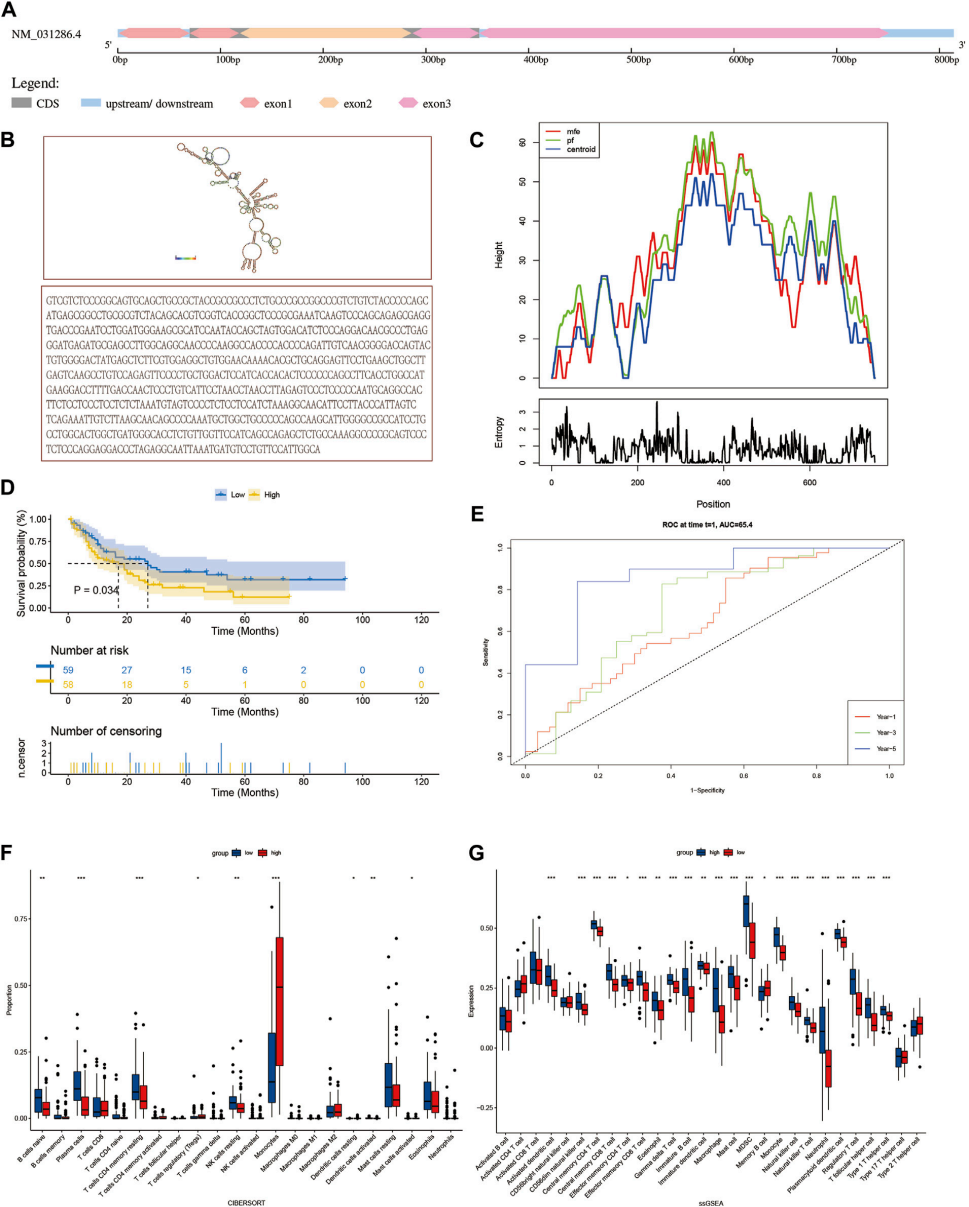
Characteristics and related biological functions of SH3BGRL3 gene: **(A)** The schematic diagram of human SH3BGRL3 genome locus. **(B)** SH3BGRL3 sequence and SH3BGRL3 secondary structure obtained from RNA fold web server (http://rna.tbi.univie.ac.at/cgi-bin/RNAWebSuite/RNAfold.cgi). **(C)** Mountain plot of MFE structure of gene SH3BGRL3. **(D)** Survival curve based on the expression of gene SH3BGRL3. **(E)** Time-related ROC curve (select 1, 3, and 5 years survival prediction variable SH3BGRL3 expression). **(F,G)** To explore the correlation between gene SH3BGRL3 expression and ciber, ssGSEA was used in quantifying immune infiltration.

The SH3 domain-binding glutamate-rich (SH3BGR) gene family consists of SH3BGR, SH3BGRL, SH3BGRL2, and SH3BGRL3. In humans, SH3BGRL3 is 751 bp in length, located on 1p36.11, and consists of three exons. The spatial secondary structure of SH3BGRL3 was predicted using RNA fold web server (http://rna.tbi.univie.ac.at/cgi-bin/RNAWebSuite/RNAfold.cg) (Figure 4A–C) (Shi et al., 2016). To further study the role of SH3BGRL3 in acute myeloid leukocytes, we performed gene expression profiling interactive analysis (GEPIA). (Supplementary Figure S1D) (Gao et al., 2017) Verifying the differential expression of SH3BGRL3 in AML patient samples, GEPIA histogram visualization showed that SH3BGRL3 was significantly overexpressed in patients with AML compared with healthy normal subjects. Kaplan–Meier survival curve showed that high SH3BGRL3 gene expression was associated with poor overall survival in patients with AML (Figure 4D). Combined with the results of time-related ROC curve evaluation (Figure 4E), the results indicated that the upregulation of SH3BGRL3 gene is involved in the progression of AML. Gene set enrichment analysis (GSEA) was used in annotating biological function and identifying the potential enrichment pathways of patients with AML and highly expressed SH3BGRL3 gene. Related functional networks were mainly involved in cell activation and cellular immune response, particularly in regulating the activation and immune function of myeloid leukocytes (Supplementary Figure S1E). (Qin et al., 2022). ssGSEA and cibersort were used in quantifying the relative infiltration level of various immune cell subsets and exploring the relationship between predictive features and immune status. The results showed that B, naïve plasma, T, CD4 memory resting, resting NK, and other immune-related cells were significantly downregulated, revealing the biological mechanism in which genes may be involved in tumor immune microenvironment (Figure 4F, G).

### Verification and identification of circSH3BGRL3 in acute myeloid leukemia

**FIGURE 5:**
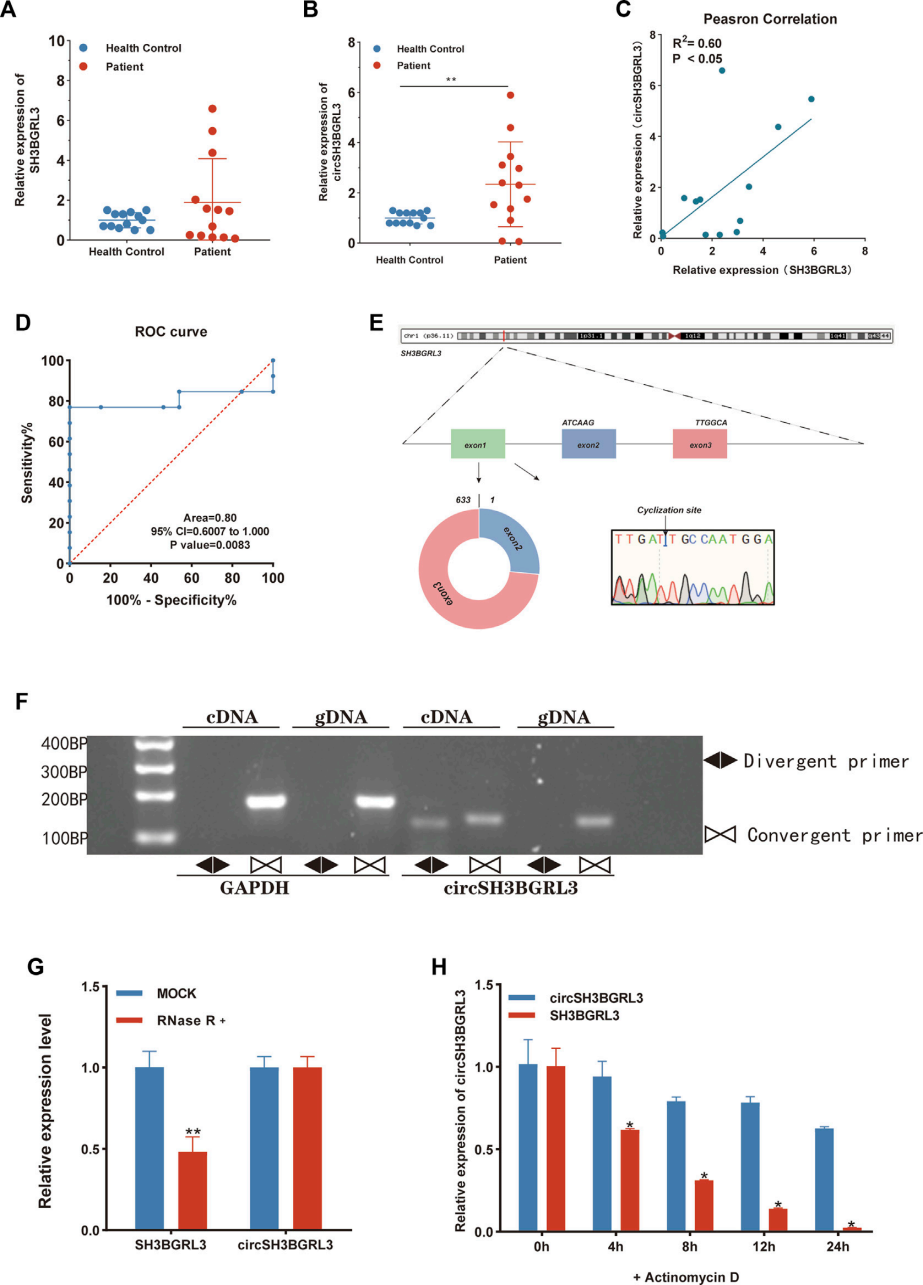
Verification and identification of circSH3BGRL3 in acute myeloid leukemia. **(A)** Real-time quantitative polymerase chain reaction (qRT-PCR) was used in determining the expression of SH3BGRL3 in 13 patients with AML and 13 healthy controls. **(B)** The expression level of circSH3BGRL3 between patients with AML (*n* = 13) and healthy controls (*n* = 13). **(C)** The expression of SH3BGRL3 was observed in the peripheral blood leukocytes of patients with AML (*n* = 13). The expression level of circSH3BGRL3 was positively correlated with the expression level of circSH3BGRL3 (R = 0.60, *p* < 0.05). **(D)** ROC analysis of circSH3BGRL3 in the peripheral blood leukocytes of patients with AML. The AUC used in distinguishing patients from normal controls was 0.80, *p* = 0.008. **(E)** Structure map of circSH3BGRL3, and the splicing junction was verified by Sanger sequencing.**(F)** Different primers were designed using the software “Primer Premier 5.00,” and divergent primers were used in amplifying circSH3BGRL3 in cDNA rather than genomic DNA (gDNA). Convergent primers amplified SH3BGRL3 and linear GAPDH RNA. **(G)** qRT-PCR analysis of circSH3BGRL3 and SH3BGRL3 mRNA abundance in AML cells treated with RNase R In cDNA and gDNA. **(H)** AML cells treated with actinomycin D were subjected to qRT-PCR analysis at 4, 8, 12, and 24 h. Measurement of circSH3BGRL3 and SH3BGRL3 mRNA abundance.

To study the differential expression of SH3BGRL3 and circSH3BGRL3 between primary AML patients and normal samples, we collected and used clinical blood samples from 13 AML patients and 13 normal individuals (controls) for qRT-PCR analysis (Figures 5A, B). The clinicopathological features of patients was shown in Table 1. We found sustained and significant increases in SH3BGRL3 and circSH3BGRL3 expression in primary AML patient samples compared to healthy controls. We analyzed the correlation between the expression of SH3BGRL3 and the expression of circSH3BGRL3 (Figure 5C). The correlation coefficient showed that the expression of circSH3BGRL3 and SH3BGRL3 mRNA was positively correlated in the clinical blood samples of patients with AML and normal individuals (controls) (Figure 5D). Next, we verified the existence of circSH3BGRL3 (has-circ-0010984) in the circBase database (Petar and Nikolaus, 2014) and the circBank database (Yang and Ding, 2019). The genome structure showed that circSH3BGRL3 is located in chr1:26607255–26608013 strand:+ and contain two relatively large second exons (633 bp) (Figure 5E), which were derived from the SH3BGRL3 gene. To further characterize circSH3BGRL3, we designed primers in different directions to amplify transcripts and used convergent and divergent primers to detect linear transcripts in cDNA and gDNA. The PCR results showed that divergent primers did not amplify circular products in gDNA but amplified circular products in cDNA. Convergent primers amplified linear products in cDNA and gDNA (Figure 5F). Then, we studied the stability of circSH3BGRL3 in AML cells. Cells were harvested at different time points after treatment with actinomycin D (a transcription inhibitor), and total RNA was isolated and extracted. The analysis of circSH3BGRL3 and SH3BGRL3 mRNAshowed that the circRNA subtype was highly stable, and the resistance of exonuclease RNase R to digestion confirmed that circSH3BGRL3 was round (Figures 5G, H). In summary, we confirmed that circSH3BGRL3 is a stable circRNA expressed in human AML cells.

**TABLE 1:** The relationship between the expression of circSH3BGRL3 and various clinicopathological variables.

Characteristics	Total (*N* = 26)	circSH3BGRL3 expression (High = 12,Low = 14)	*p*-value*
Gender			
Male	9	7	*p* = 0.019*
female	17	5	
Age			
≥60	5	4	*p* = 0.091
<60	21	8	
State			
Patient	13	11	*p* < 0.001***
health	13	1	
No recurrence			
Yes	1	1	*p* = 0.657
No	12	10	

*p < 0.05, ***p < 0.001, ****p < 0.0001.

### Overexpression of circSH3BGRL3 promotes cell proliferation and accelerates cell cycle in AML

**FIGURE 6:**
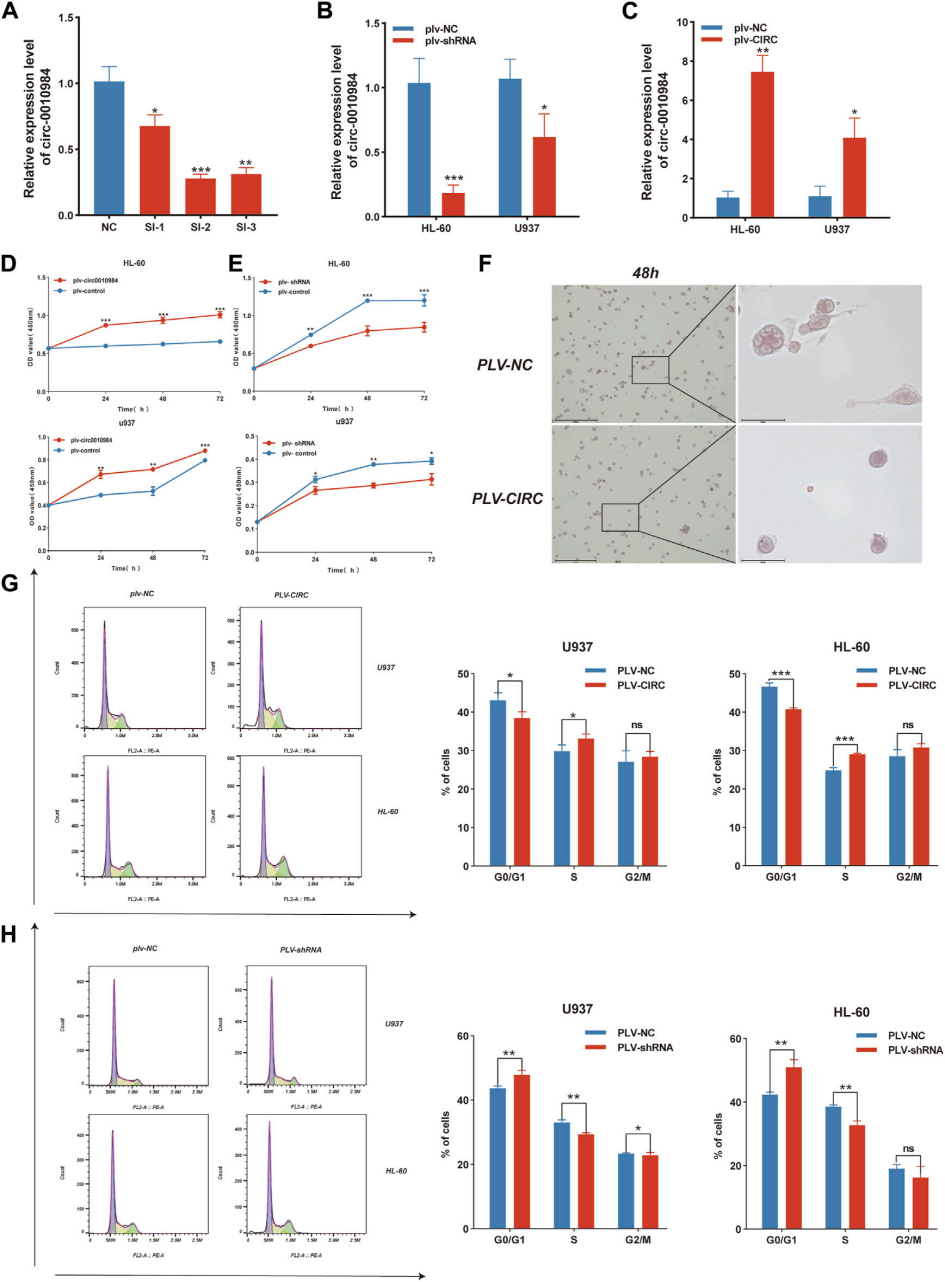
CircSH3BGRL3 promotes the proliferation of AML cells and accelerates the cell cycle. **(A)** Three siRNA molecules with different structures were designed for circ-0010984 and transfected into cells to verify the knockdown effect (*p < 0.05, **p < 0.01, ***p < 0.001). **(B)** Construction of siRNA plasmid vector. The expression level of circ_0010984 in U937 and HL-60 cells treated with si-circ_0010984 and si-NC was detected by real-time quantitative polymerase chain reaction (qRT-PCR) after lentivirus packaging infection (*p < 0.05, ***p < 0.001). **(C)** The overexpression plasmid vector of circ-0010984 was constructed. The expression levels of circ_0010984 in U937 and HL-60 cells were detected by qRT-PCR after lentivirus packaging infection (*p < 0.05, **p < 0.01). **(D)** CCK-8 assay was used to analyze the effect of circ_0010984 overexpression on the viability of U937 and HL-60 cells. **(E)** The effect of circ_0010984 knockdown on si-circ_0010984-treated U937 and HL-60 cells was evaluated by CCK-8 assay. **(F)** After THP-1 cells were induced to differentiate, Cell morphology was observed under a microscope (scale bar = 50 μm). Use Swiss–Giemsa staining. **(G)** Cell cycle analysis of U937 and HL-60 cells after transfection with overexpression vector and control vector. **(H)** Cell cycle analysis of U937 and HL-60 cells subjected to plv-sh-circ_0010984 and plv-sh-NC transfection.

To evaluate whether circSH3BGRL3 affects the progression of leukemia, we constructed a vector (pLV-circRNA0010984) that effectively expresses circSH3BGRL3 in AML cell lines (Supplementary Figure S2A). Three groups of small interfering RNA molecules targeting circSH3BGRL3 were used for circ-SH3BGRL3 silencing, and si-NC was used as a control. To verify the knockdown efficiency, we detected the expression of circSH3BGRL3 in transfected cells. The second group of small interfering RNA (si-hsa-circ-0010984-002) was the most effective in down-regulating the endogenous expression of circSH3BGRL3 and more effective than si-NC (Figure 6A), so we selected the second group to construct the vector (PLVX-SHRNA2-PURO) for lentivirus packaging (Supplementary Figures S2B, C). To determine the efficiency of overexpression and knockdown, we performed qRT-PCR analysis and detected the expression of circSH3BGRL3 in transfected cells (Figures 6B, C). The siRNAs and overexpression plasmid affected the expression of circSH3BGRL3, but not the linear SH3BGRL3 mRNA (Supplementary Figures S2D–F). The overexpression of circSH3BGRL3 significantly accelerated the proliferation of AML cells (Figures 6D, E). Meanwhile, the overexpression of circSH3BGRL3 affected THP-1 cell differentiation, inducing less-lobulated nuclei to exhibit immature appearance (Figure 6F). Monocyte differentiation was assessed by observing cell morphology on the slides and detecting macrophage surface molecular marker CD86 by FACS analysis (Supplementary Figure S3A). In addition, the upregulation of circSH3BGRL3 significantly reduced the percentage of G0/G1 phase cells and increased the percentage of S phase cells. Compared with pLV-circSH3BGRL3-NC control transfection, the overexpression of circSH3BGRL3 accelerated the cell cycle (Figures 6G, H). As for the knockdown group, the cells transfected with si-circSH3BGRL3 proliferated slowly, and the cell division cycle was slowed down. These data showed the functional relevance of circSH3BGRL3 in AML.

### CircSH3BGRL3 silencing reduces resistance of AML cells to DAU

**FIGURE 7:**
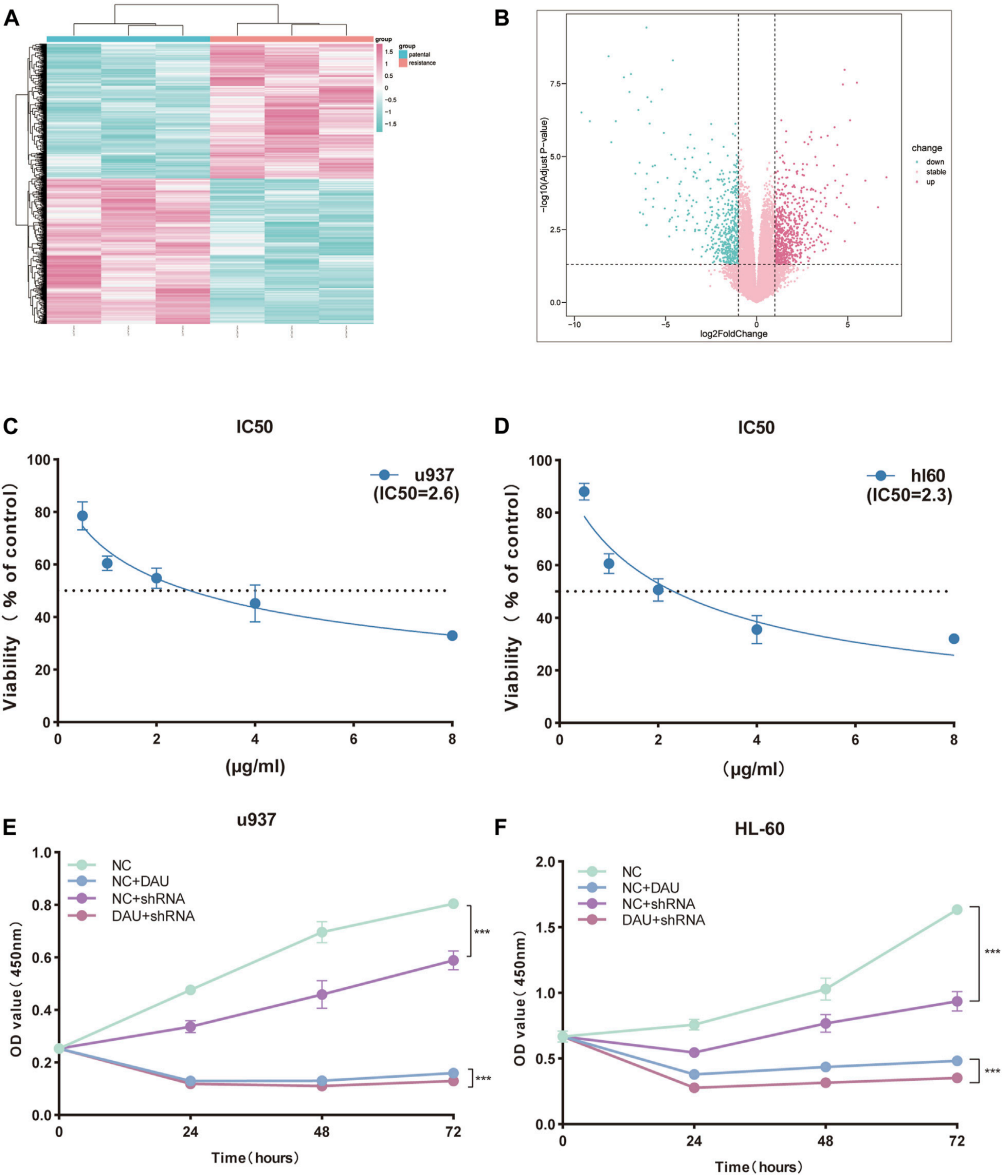
CircSH3BGRL3 Silencing Reduces Resistance of AML Cells to DAU. **(A)** For the GSE193094 dataset, the differentially expressed mRNA molecules of daunorubicin-resistant cell lines were presented in the form of a heat map (*p* < 0.05). **(B)** The differentially expressed mRNA molecules were presented in the form of a volcano map (|log2FoldChange| < 1 indicates no expression difference), and the *p* values of the differentially expressed genes were sorted from small to large. **(C)** CCK-8 was used in determining the half-inhibitory concentration of daunorubicin on U937 cells. **(D)** Determination of half-inhibitory concentration. **(E)** Determination of circ_0010984 knockdown by CCK-8 and the effect of combination drug daunorubicin on U937 cell viability. **(F)** Daunorubicin on HL-60 cells by CCK-8 determination of circ_0010984 knockdown and the effect of combination drug daunorubicin on HL-60 cell viability.

DA regimen is mainly used to treat AML and composed of DAU and cytarabine. We studied the function of circSH3BGRL3 in DAU-treated AML cells to observe the role of circSH3BGRL3 in combined clinical drug therapy (Supplementary Figure S3B) (Tracy and Murphy, 2017). First, apart from using bioinformatics, we designated nine cell sample groups from GSE193094 as parental HL-60, HRA, and HRD. To screen genes involved in DAU resistance, we analyzed the differentially expressed genes (DEGs) between parental HL-60 and HRD (Figures 7A, B). SH3BGRL3 was identified as one of the dysregulated mRNA molecules between parental HL-60 and HRD. The expression of SH3BGRL3 in DAU-resistant AML cells significantly increased relative to that in normal AML cells (Supplementary Figure S3C), indicating that SH3BGRL3 plays a role in DAU drug tolerance mechanism. Given that the concentration of DAU is unknown, we first screened the IC50 of DAU on different AML cells (Figures 7C, D). The silencing of circSH3BGRL3 affected the resistance of AML cells to DAU. Compared with the knockout of circSH3BGRL3 alone, AML cells treated with DAU after the knockout of circSH3BGRL3 showed more obvious proliferation arrest (Figures 7E, F). circSH3BGRL3 silencing inhibited the activity of AML cells and enhanced the sensitivity of AML cells to DAU.

### CircSH3BGRL3 acts as a sponge for mir-375-3p

**FIGURE 8:**
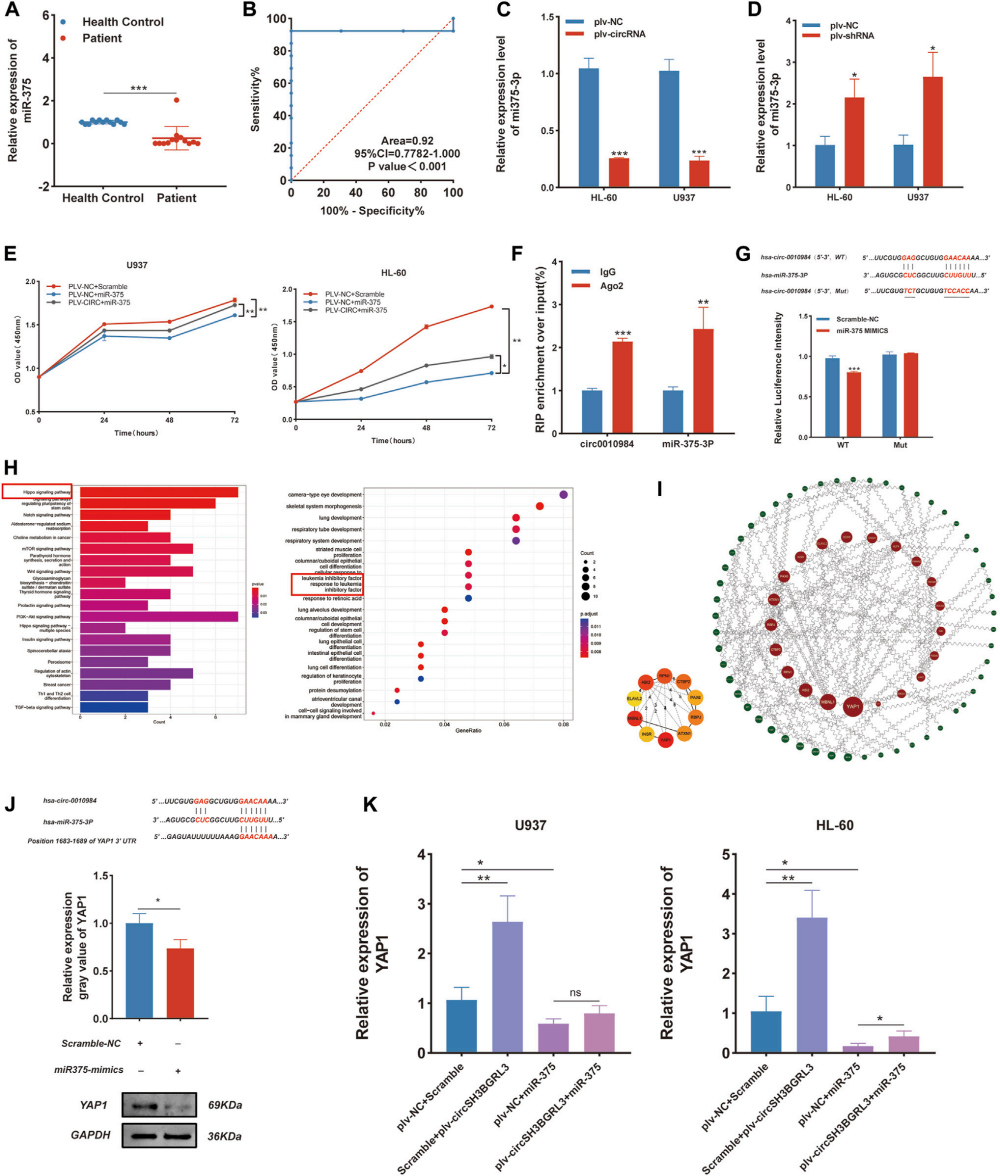
CircSH3BGRL3 Acts as a Sponge for miR-375-3P. **(A)** Real-time quantitative polymerase chain reaction (qRT-PCR) was used in determining the expression of miR-375 in 13 patients with AML and 13 healthy controls. **(B)** ROC analysis of miR-375 in the peripheral blood leukocytes of patients with AML. The AUC used in distinguishing patients from normal controls was 0.92, *p* < 0.001. **(C)** The relative levels of miR-375 in HL-60 and U937 cells transfected with plv-NC or plv-circSH3BGRL3 were measured by real-time quantitative polymerase chain reaction (qRT-PCR). **(D)** The relative level of miR-375 in HL-60 and U937 cells transfected with plv-NC or plv-shRNA was measured by qRT-PCR (*p < 0.05). **(E)** Cell proliferation decreased upon miR-375 overexpression and was rescued after circRNA_0010984 overexpression in U937 and HL-60 cells.**(F)** RIP experiments were performed using anti-Ago2 antibodies in AML cell lysates, and anti-IgG antibodies were selected as negative controls. **(G)** Dual-luciferase reporter assay was utilized to verify the interaction between circ_0010984 and miR-375. **(H)** GO and KEGG functional enrichment analysis of miR-375. **(I)**Regulated by miR-375 from the protein-protein interaction network. **(J)** Inferred binding site sequences between circ_0010984 and miR-375 and between miR-375 and YAP1. Transfection mimics were used in detecting the relative expression of YAP1 in AML cells with Western blot assay (*p < 0.05). **(K)**Relative expression of YAP1 was measured by qRT-PCR in circ_0010984 and miR-375 mimics treated U937 cells and HL-60 cells.

CircRNA usually interacts with miRNA. Hence, we used circRNA interactome (https://circinteractome.nia.nih.gov/) to predict candidate miRNA molecules that interact with circSH3BGRL3. The results showed that twenty-three miRNAs might act as targets of circ_0010984 (Supplementary Table S2). We then analyzed the impact of all miRNAs on patient survival status through the Oncolnc database. The results showed that Oncolnc database could only retrieve three miRNAs (miR-375, miR-338-3p, and miR-940) containing prognostic information (Supplementary Figures S4A, B). The expression of only one miRNA (miRNA-375) is beneficial and significantly correlated with the survival status of patients. We analyzed the expression level of miRNA-375 in healthy controls and AML patients (qRT-PCR) (Figure 8A), and the results revealed that miRNA-375 expression was markedly increased in healthy controls relative to AML patients (Figure 8B). We found that circSH3BGRL3 has a binding site that regulates miR-375, which is a conserved noncoding RNA involved in tumor cell proliferation and migration and drug resistance (Xu et al., 2021). The upregulation of miR-375 is related to the poor prognosis of AML in children and is a potential biomarker for alleviating AML in pediatric patients (Feng and Weijing, 2013). We chose miR-375 for further study. We analyzed the correlation between the expression levels of miR-375 and circSH3BGRL3 (Supplementary Figure S4C). The overexpression of circSH3BGRL3 reduced the content of miR-375 in AML cells. By contrast, the knockdown of circSH3BGRL3 increased the content of miR-375 in AML cells (Figures 8C, D). Furthermore, the mimics of miR-375 significantly attenuated the proliferation which induced by enhancing circ_0010984 expression in U937 and HL-60 cells (Figure 8E). The results of RNA immunoprecipitation experiments further confirmed that circSH3BGRL3 has the binding site of Ago2 protein, which can be enriched to miR-375. In the dual-luciferase reporter assay, we found that miR-375 mimics could significantly decrease the luciferaseactivity of cancer cells driven by circ_0010984-WT, but not that driven by circ_0010984-Mut, compared with the scramble group (Figures 8F, J). We explored the biological functions and metabolic pathways of miR-375 and performed GO and KEGG functional enrichment (Figure 8H). Subsequently, we analyzed the specific target mRNA of miR-375 and used the STRING (functional protein association networks) database to construct a protein–protein interaction network (Supplementary Table S3), and miRNA–mRNA regulatory sites were queried using Targetscan (www.targetscan.org), microT (www.biostars.org). Then, we used ClueGo to visualize the enrichment pathway (Supplementary Figures S4D, E). The Cytoscape plug-in cytoNCA was used in screening the most reliable core genes of betweenness centrality. The results showed that YAP1 played the most significant role in the regulatory network of protein interactions (Figure 8I). Western blot results showed that YAP1 can be used as a downstream target regulated by miR-375, and miR-375 treatment resulted in the significant downregulation of YAP1 protein in cells (Figure 8J). In addition, we demonstrated that circ_0010984 treatment resulted in a significant upregulation of YAP1, and miR-375 treatment resulted in a remarkable downregulation of YAP1 in U937 cells and HL-60 cells. The miR-375 induced downregulation of YAP1 could be reversed by circ_0010984 (Figure 8K).

## Discussion

Although some breakthroughs have been made in the treatment and diagnosis of AML in recent years, it is still one of the most lethal hematological malignancies due to its complex genetic and molecular mechanisms. AML treatment with DAU and cytarabine is a common method for patients. Although the application of drugs is expected to overcome the disease, a large number of clinical data have shown that the treatment effect worsens with increasing treatment duration. Therefore, fully understanding the molecular mechanisms involved in the occurrence and development of AML and determining novel prognostic treatment options are important. Wang et al. (Yanjun et al., 2021) explored the relationship between genes and AML and demonstrated that ferroptosis-related genes play an important role in the development of AML. Laura et al. (Hueneman et al., 2022) proposed that blocking UBE2N can eliminate the carcinogenic immune signal of AML. However, whether these target genes and their signaling pathways can be successfully applied to clinical practice and exert clinical efficacy requires long-term clinical experiments and exploration. The purpose of this study is to identify genes with diagnostic and prognostic values and to explore novel diagnostic and therapeutic options for AML patients.

In the current study, we found that some genes in patients with AML that can be used in predicting the prognoses of patients by constructing a risk model. We found that the SH3BGRL3 gene, which may produce circRNA by exon cyclization, regulates downstream genes and affects the related functions of diseases through molecular sponge mechanism. To study SH3BGRL3, Yin et al. (Wang et al., 2019) presented evidence that SH3BGRL3 can promote the growth and metastasis of renal clear cell carcinoma. Nie et al. (Wei et al., 2021) demonstrated that the high expression of SH3BGRL3 is related to the poor prognosis of glioblastoma. These results indicate that SH3BGRL3 is closely related to the diagnosis and prognosis of various malignant proliferative diseases. SH3BGRL3 is associated with the malignant proliferation of tumors and is involved in tumor progression in many different types of cancer.

In this study, we found a possible exon-cycling RNA of SH3BGRL3:circRNA_0010984. circRNA is a noncoding RNA with a closed circular single-stranded structure and currently at the forefront of tumor molecular biology research. The expression of circRNA is tissue specific and highly conserved. Many related studies have reported that circRNA is closely related to the occurrence and development of AML. CircNFIX can affect the tumorigenicity of AML by targeting the miR876-3P/TRIM31 axis (Wu et al., 2022). circ_0015278 can regulate AML progression through ferroptosis-related genes (Yang et al., 2022).

In this experiment, we hypothesized that circRNA_0010984 has a certain degree of correlation with the transcriptional expression of the host linear gene SH3BGRL3. To verify our hypothesis, we first examined the coexpression relationship between SH3BGRL3 and circRNA_0010984. The results showed that the expression level of circRNA_0010984 was positively correlated with the expression level of its host linear gene SH3BGRL3. We further verified the function of circRNA_0010984 in AML cell lines. Knocking out circ-0010984 can effectively inhibit the proliferation of AML cells. Therefore, we believe that the abnormal expression level of SH3BGRL3 transcript is closely related to AML, and the upregulation of circ-0010984 can promote the malignant proliferation of AML.

Our study aims to identify the key DEGs associated with AML, establish a prognostic model, and link gene expression with patient prognosis. Bioinformatics data involved in this study come from different bioinformatics platforms (including TCGA database). To eliminate errors as much as possible, we deleted missing data. Lasso regression has advantages over univariate analysis and can solve the problem of multicollinearity between variables. In this study, we established a nomogram based on the Lasso-Cox regression model, which has certain reference value for analyzing the risk of multiple different variables affecting patients. The prognostic feature model constructed by Kaplan–Meier survival curve and ROC model fully guaranteed the credibility of the results. We then constructed a ceRNA regulatory network to determine the regulatory relationship between the diagnosis and prognosis of AML. It was reliably confirmed in the statistical analysis of the clinical samples.

To better explore the potential mechanism of circRNA_0010984 and determine whether circRNA_0010984 plays its biological function as a miRNA sponge. We speculate that in addition to Ago2 protein, EIF4A3, FUS and SFRS1 can also be used as RNA-binding proteins to match circRNA_0010984. The experimental data proved that miR-375 is a key downstream effector of circRNA_0010984 in our model. Many studies have confirmed that miR-375 interacts with a large number of target genes and participates in regulating many physiological processes related to human diseases (MatveevaBaulina et al., 2022). The downregulation of miR-375 contributes to ERBB2-mediated VEGFA overexpression in esophageal cancer (Ren et al., 2021). Although miR-375 is a key regulator of human cancer progression, studies on the function and mechanism of AML are few. The regulation of the hippo signaling pathway can affect cell proliferation, division, and death. When the pathway is abnormally inhibited, the tissues and organs of animals will proliferate excessively and cause tumors. Through GO and KEGG functional enrichment analysis, we confirmed the key role of miR-375 in regulating target gene YAP1. miRNA regulates the expression of target mRNA through complementary sequences in the 3′-untranslated region. Finally, we studied the relationship between miR-375 and YAP1 in leukemia cells. Western blot results showed that YAP1 expression was negatively correlated with miR-375 expression.

## Conclusion

This study identified the upregulated expression of the hub gene SH3BGRL3 in AML, and we predicted the targeting effect of circ_0010984. The ceRNA network obtained from bioinformatics analysis and its potential function of binding to target genes can be confirmed in future experiments. These data suggest that circRNA_0010984 and its parent gene SH3BGRL3 are potential therapeutic targets for AML.

## Data Availability

Publicly available datasets were analyzed in this study. This data can be found here, NCBI Gene Expression Omnibus (GSE193094,GSE17054).
