# Supplementary figures and images for: Gene SH3BGRL3 regulates acute myeloid leukemia progression through circRNA_0010984 based on competitive endogenous RNA mechanism
Source: Front Cell Dev Biol. 2023 Jun 12;11:1173491. doi: 10.3389/fcell.2023.1173491 (PMC10313326; doi:10.3389/fcell.2023.1173491)

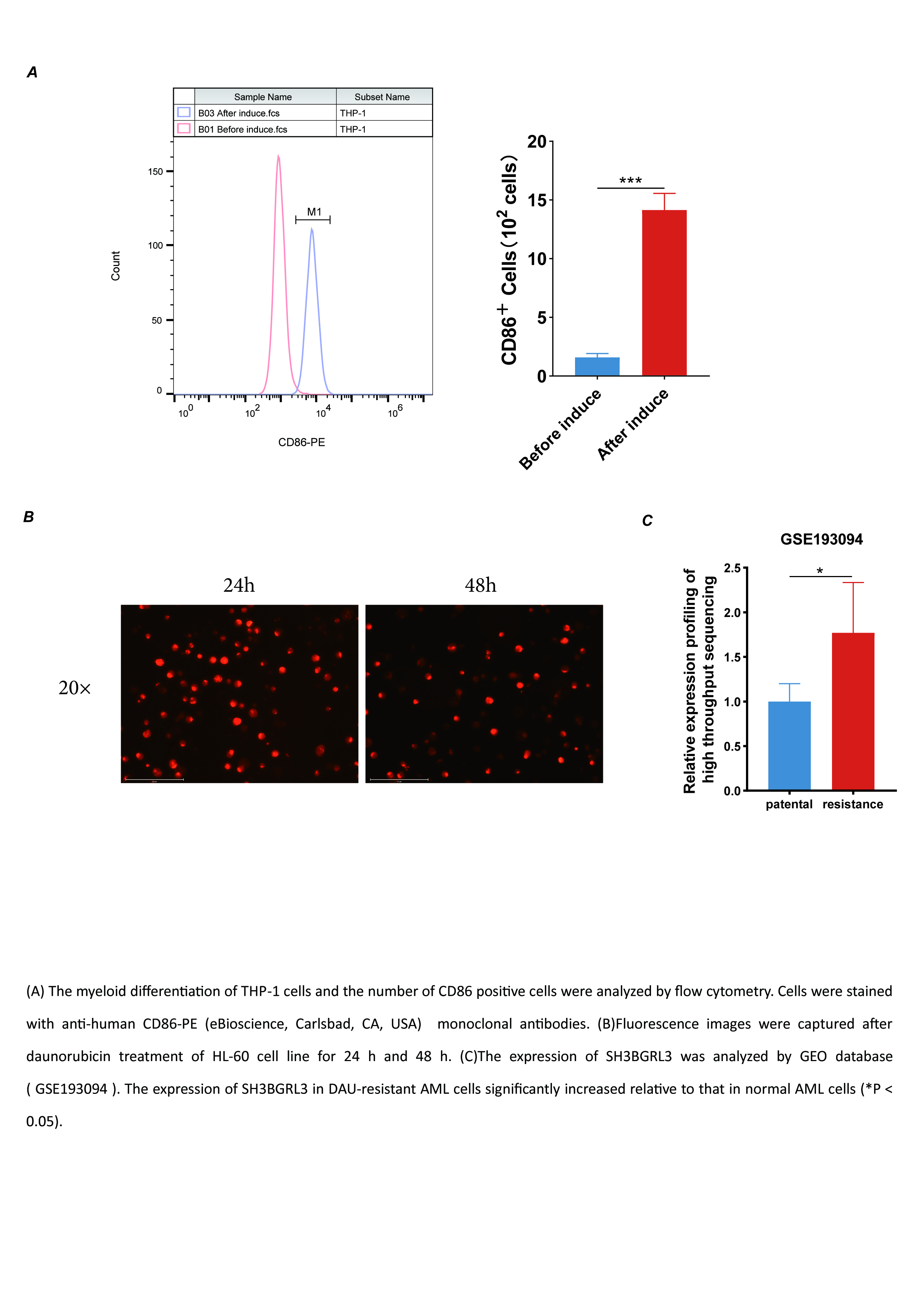

Supplement: Supplementary file 1 [file Image3.jpeg]

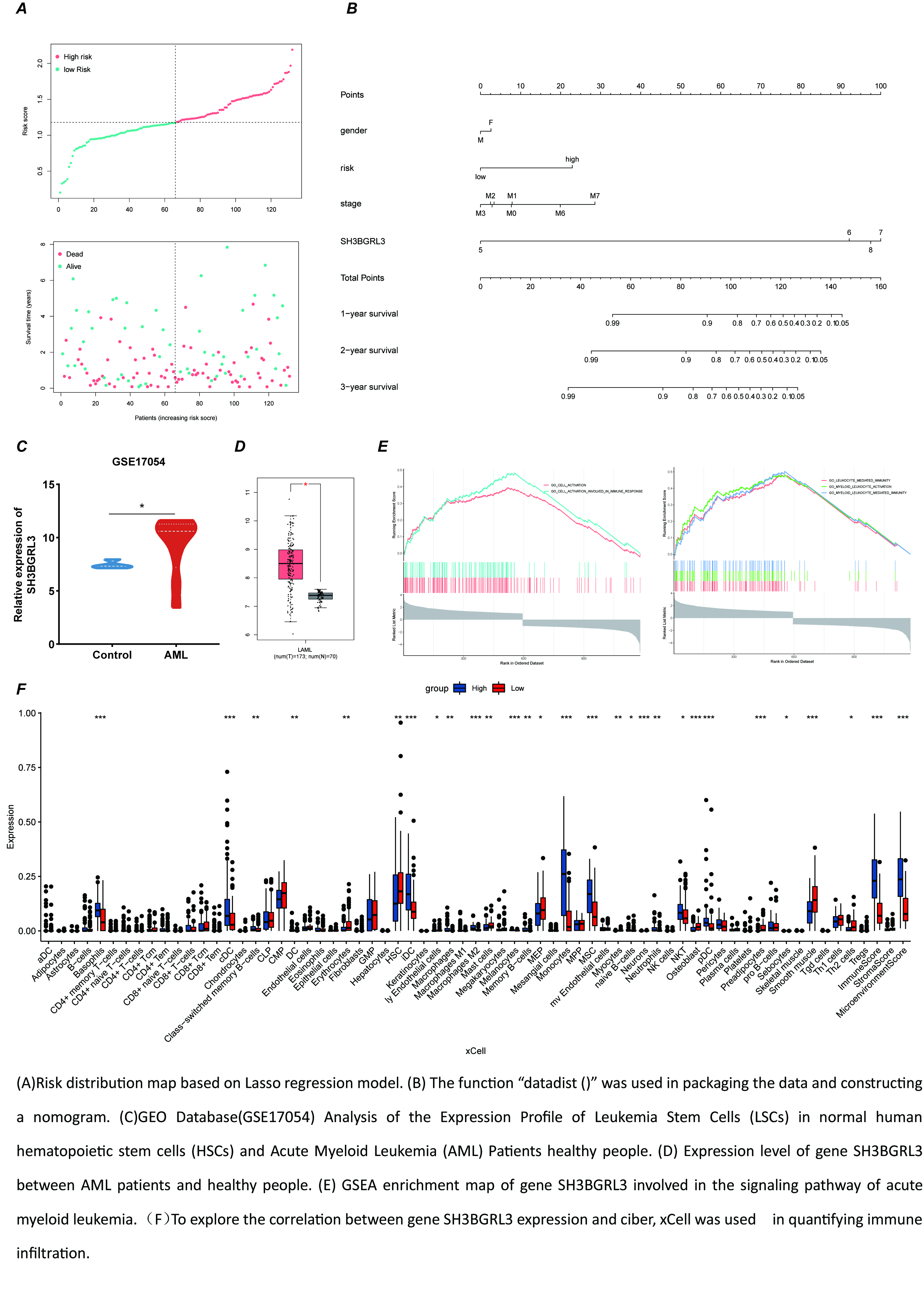

Supplement: Supplementary file 3 [file Image1.jpeg]

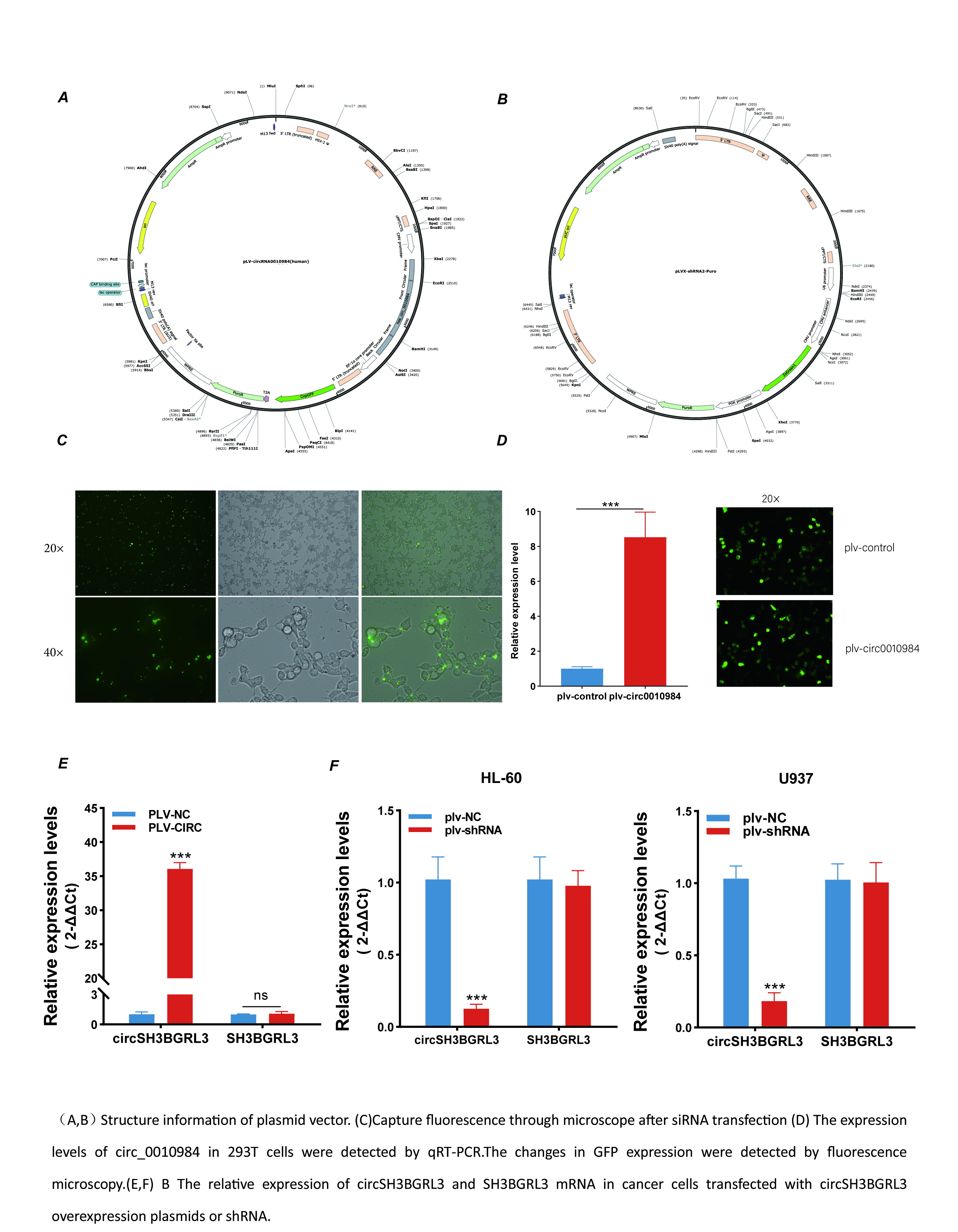

Supplement: Supplementary file 4 [file Image2.jpeg]

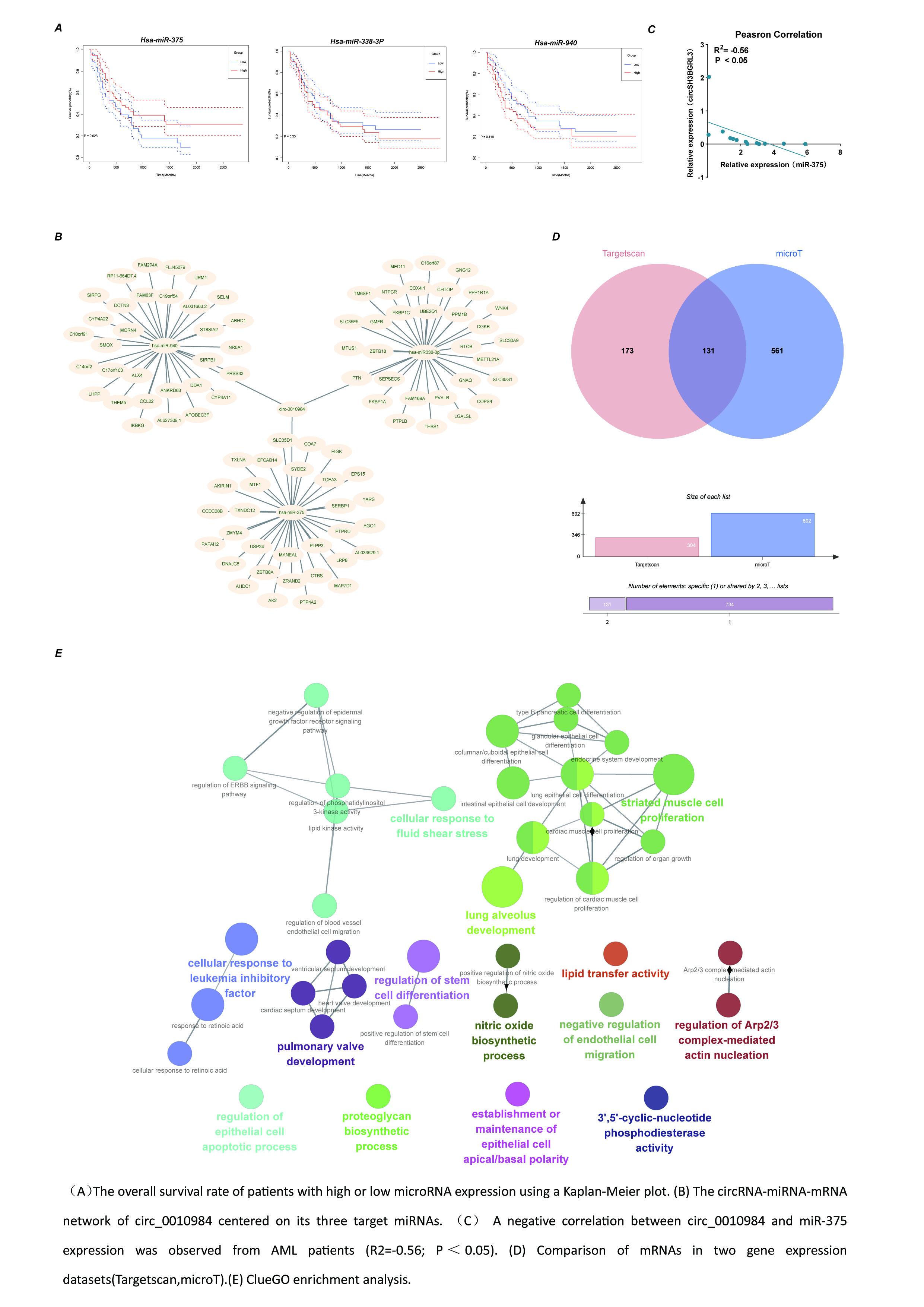

Supplement: Supplementary file 7 [file Image4.jpg]
