# Supplementary material for: Gene SH3BGRL3 regulates acute myeloid leukemia progression through circRNA_0010984 based on competitive endogenous RNA mechanism
Source: Front Cell Dev Biol. 2023 Jun 12;11:1173491. doi: 10.3389/fcell.2023.1173491 (PMC10313326; doi:10.3389/fcell.2023.1173491)
Supplement: Supplementary file 5 [file Table2.docx]

**Supplementary Table s2 Target miRNAs of circ_0010984 predicted by circinteractome.**

| CircRNA Mirbase ID | CircRNA (Top) - miRNA (Bottom) pairing | Site Type | CircRNA Start | CircRNA End |
| --- | --- | --- | --- | --- |
| [hsa_circ_0010984](http://www.circbase.org/cgi-bin/singlerecord.cgi?id=hsa_circ_0010984) (5' ... 3')  [hsa-miR-1182](http://www.mirbase.org/cgi-bin/mirna_entry.pl?acc=hsa-miR-1182) (3' ... 5') | UCCCUCUCCCAGGAG-GACCCUAG       \|\|\|\|      \|\|\|\|\|\|   CAGUGUAGGGAGGGUUCUGGGAG | 7mer-1a | 594 | 600 |
| [hsa_circ_0010984](http://www.circbase.org/cgi-bin/singlerecord.cgi?id=hsa_circ_0010984) (5' ... 3')  [hsa-miR-1184](http://www.mirbase.org/cgi-bin/mirna_entry.pl?acc=hsa-miR-1184) (3' ... 5') | UGUGGAACAAAACACGCUGCAGG                  \|\|\|\|\|\|\|    CCUUCGGUAGUUCAGCGACGUCC | 7mer-m8 | 207 | 213 |
| [hsa_circ_0010984](http://www.circbase.org/cgi-bin/singlerecord.cgi?id=hsa_circ_0010984) (5' ... 3')  [hsa-miR-1258](http://www.mirbase.org/cgi-bin/mirna_entry.pl?acc=hsa-miR-1258) (3' ... 5') | CCAACUCCCUGUCAUUCCUAACC                  \|\|\|\|\|\|\|      AAGGUGCUGGAUUAGGAUUGA | 7mer-m8 | 332 | 338 |
| [hsa_circ_0010984](http://www.circbase.org/cgi-bin/singlerecord.cgi?id=hsa_circ_0010984) (5' ... 3')  [hsa-miR-1278](http://www.mirbase.org/cgi-bin/mirna_entry.pl?acc=hsa-miR-1278) (3' ... 5') | AUUGUCAACGGGGACCAGUACUG                \|\|\|\|\|\|\|   UAUCUACUAUACGUGUCAUGAU | 7mer-m8 | 157 | 163 |
| [hsa_circ_0010984](http://www.circbase.org/cgi-bin/singlerecord.cgi?id=hsa_circ_0010984) (5' ... 3')  [hsa-miR-1288](http://www.mirbase.org/cgi-bin/mirna_entry.pl?acc=hsa-miR-1288) (3' ... 5') | UCUGCCAAAGGCCCCGCAGUCCC                \|\|\|\|\|\|\|    AGAGGUCUAGUCCCGUCAGGU | 7mer-m8 | 575 | 581 |
| [hsa_circ_0010984](http://www.circbase.org/cgi-bin/singlerecord.cgi?id=hsa_circ_0010984) (5' ... 3')  [hsa-miR-1289](http://www.mirbase.org/cgi-bin/mirna_entry.pl?acc=hsa-miR-1289) (3' ... 5') | CAGAGUUCCCCUGCUGGACUCCA                      \|\|\|\|\|\|\|        UUUUACGUCUAAGGACCUGAGGU | 8mer-1a | 262 | 269 |
| [hsa_circ_0010984](http://www.circbase.org/cgi-bin/singlerecord.cgi?id=hsa_circ_0010984) (5' ... 3')  [hsa-miR-1303](http://www.mirbase.org/cgi-bin/mirna_entry.pl?acc=hsa-miR-1303) (3' ... 5') | ACUUCUCCUCCCUCCUCUCUAAA                \|\|\|\|\|\|\|   UCUCGUUCUGGGGCAGAGAUUU | 8mer-1a | 386 | 393 |
| [hsa_circ_0010984](http://www.circbase.org/cgi-bin/singlerecord.cgi?id=hsa_circ_0010984) (5' ... 3')  [hsa-miR-149](http://www.mirbase.org/cgi-bin/mirna_entry.pl?acc=hsa-miR-149) (3' ... 5') | UCUGUUGGUUCCAUCAGCCAGAG                 \|\|\|\|\|\|   CCCUCACUUCUGUGCCUCGGUCU | 7mer-1a | 551 | 557 |
| [hsa_circ_0010984](http://www.circbase.org/cgi-bin/singlerecord.cgi?id=hsa_circ_0010984) (5' ... 3')  [hsa-miR-1825](http://www.mirbase.org/cgi-bin/mirna_entry.pl?acc=hsa-miR-1825) (3' ... 5') | CCGCCAUCCUGCCUGGCACUGGC                \|\|\|\|\|\|\|       CCUCUCCUCCCGUGACCU | 7mer-m8 | 517 | 523 |
| [hsa_circ_0010984](http://www.circbase.org/cgi-bin/singlerecord.cgi?id=hsa_circ_0010984) (5' ... 3')  [hsa-miR-182](http://www.mirbase.org/cgi-bin/mirna_entry.pl?acc=hsa-miR-182) (3' ... 5') | CAUCAGCCAGAGCUCUGCCAAAG                  \|\|\|\|\|\|   UCACACUCAAGAUGGUAACGGUUU | 7mer-1a | 562 | 568 |
| [hsa_circ_0010984](http://www.circbase.org/cgi-bin/singlerecord.cgi?id=hsa_circ_0010984) (5' ... 3')  [hsa-miR-338-3p](http://www.mirbase.org/cgi-bin/mirna_entry.pl?acc=hsa-miR-338-3p) (3' ... 5') | UGUCCAGAGUUCCCCUGCUGGAC                  \|\|\|\|\|\|     GUUGUUUUAGUGACUACGACCU | 7mer-1a | 258 | 264 |
| [hsa_circ_0010984](http://www.circbase.org/cgi-bin/singlerecord.cgi?id=hsa_circ_0010984) (5' ... 3')  [hsa-miR-375](http://www.mirbase.org/cgi-bin/mirna_entry.pl?acc=hsa-miR-375) (3' ... 5') | UUCGUGGAGGCUGUGGAACAAAA       \|\|\|      \|\|\|\|\|\|   AGUGCGCUCGGCUUGCUUGUUU | 7mer-1a | 196 | 202 |
| [hsa_circ_0010984](http://www.circbase.org/cgi-bin/singlerecord.cgi?id=hsa_circ_0010984) (5' ... 3')  [hsa-miR-409-3p](http://www.mirbase.org/cgi-bin/mirna_entry.pl?acc=hsa-miR-409-3p) (3' ... 5') | UCCUCCAUCUAAAGGCAACAUUC                \|\|\|\|\|\|\|   UCCCCAAGUGGCUCGUUGUAAG | 7mer-m8 | 421 | 427 |
| [hsa_circ_0010984](http://www.circbase.org/cgi-bin/singlerecord.cgi?id=hsa_circ_0010984) (5' ... 3')  [hsa-miR-486-3p](http://www.mirbase.org/cgi-bin/mirna_entry.pl?acc=hsa-miR-486-3p) (3' ... 5') | AGCCCCAAAUGCUGGCUGCCCCC                 \|\|\|\|\|\|\|     UAGGACAUGACUCGACGGGGC | 7mer-m8 | 479 | 485 |
| [hsa_circ_0010984](http://www.circbase.org/cgi-bin/singlerecord.cgi?id=hsa_circ_0010984) (5' ... 3')  [hsa-miR-555](http://www.mirbase.org/cgi-bin/mirna_entry.pl?acc=hsa-miR-555) (3' ... 5') | CUAAAGGCAACAUUCCUUACCCA                 \|\|\|\|\|\|\|     UAGUCUCCAAGUCGAAUGGGA | 8mer-1a | 429 | 436 |
| [hsa_circ_0010984](http://www.circbase.org/cgi-bin/singlerecord.cgi?id=hsa_circ_0010984) (5' ... 3')  [hsa-miR-571](http://www.mirbase.org/cgi-bin/mirna_entry.pl?acc=hsa-miR-571) (3' ... 5') | UGAAGGACCUUUUGACCAACUCC                  \|\|\|\|\|\|\|      GAGUGAGUCUACCGGUUGAGU | 7mer-m8 | 317 | 323 |
| [hsa_circ_0010984](http://www.circbase.org/cgi-bin/singlerecord.cgi?id=hsa_circ_0010984) (5' ... 3')  [hsa-miR-574-5p](http://www.mirbase.org/cgi-bin/mirna_entry.pl?acc=hsa-miR-574-5p) (3' ... 5') | GCUGGACUCCAUCACCACACUCC                 \|\|\|\|\|\|\|   UGUGUGAGUGUGUGUGUGUGAGU | 7mer-m8 | 274 | 280 |
| [hsa_circ_0010984](http://www.circbase.org/cgi-bin/singlerecord.cgi?id=hsa_circ_0010984) (5' ... 3')  [hsa-miR-625](http://www.mirbase.org/cgi-bin/mirna_entry.pl?acc=hsa-miR-625) (3' ... 5') | UAACCUUAGAGUCCCUCCCCCAA       \|\|\|\|     \|\|\|\|\|\|   CCUGAUAUCUUGAA-AGGGGGA | 7mer-1a | 355 | 361 |
| [hsa_circ_0010984](http://www.circbase.org/cgi-bin/singlerecord.cgi?id=hsa_circ_0010984) (5' ... 3')  [hsa-miR-637](http://www.mirbase.org/cgi-bin/mirna_entry.pl?acc=hsa-miR-637) (3' ... 5') | CCCAAGGCCACCCCACCCCAGAU                  \|\|\|\|\|\|   UGCGUCUCGGGCUUUCGGGGGUCA | 7mer-1a | 136 | 142 |
| [hsa_circ_0010984](http://www.circbase.org/cgi-bin/singlerecord.cgi?id=hsa_circ_0010984) (5' ... 3')  [hsa-miR-647](http://www.mirbase.org/cgi-bin/mirna_entry.pl?acc=hsa-miR-647) (3' ... 5') | AAUGCUGGCUGCCCCCAGCCAAG                \|\|\|\|\|\|    CUUCCUUCACUCACGUCGGUG | 7mer-1a | 486 | 492 |
| [hsa_circ_0010984](http://www.circbase.org/cgi-bin/singlerecord.cgi?id=hsa_circ_0010984) (5' ... 3')  [hsa-miR-665](http://www.mirbase.org/cgi-bin/mirna_entry.pl?acc=hsa-miR-665) (3' ... 5') | GCGAGGUGACCCGAAUCCUGGAU                  \|\|\|\|\|\|       UCCCCGGAGUCGGAGGACCA | 7mer-1a | 32 | 38 |
| [hsa_circ_0010984](http://www.circbase.org/cgi-bin/singlerecord.cgi?id=hsa_circ_0010984) (5' ... 3')  [hsa-miR-873](http://www.mirbase.org/cgi-bin/mirna_entry.pl?acc=hsa-miR-873) (3' ... 5') | AAACACGCUGCAGGAGUUCCUGA                \|\|\|\|\|\|\|    UCCUCUGAGUGUUCAAGGACG | 8mer-1a | 216 | 223 |
| [hsa_circ_0010984](http://www.circbase.org/cgi-bin/singlerecord.cgi?id=hsa_circ_0010984) (5' ... 3')  [hsa-miR-940](http://www.mirbase.org/cgi-bin/mirna_entry.pl?acc=hsa-miR-940) (3' ... 5') | CAUUGGGGCCGCCAUCCUGCCUG                \|\|\|\|\|\|\|    CCCCUCGCCCCCGGGACGGAA | 7mer-m8 | 509 | 515 |
|  |  |  |  |  |
